# Levelling the playing field: Exploring inequalities and exclusions with a community‐based football league for people with experience of mental distress

**DOI:** 10.1111/1440-1630.12791

**Published:** 2022-01-23

**Authors:** Anna Pettican, Ewen Speed, Wendy Bryant, Cherry Kilbride, Peter Beresford

**Affiliations:** ^1^ School of Health and Social Care University of Essex Colchester UK; ^2^ Department of Health Sciences Brunel University London Uxbridge UK

**Keywords:** football, health inequalities, mental distress, occupational marginalisation, occupational therapy, participatory action research, physical (in)activity, sport

## Abstract

**Introduction:**

Sport workforce strategy in the United Kingdom (UK) has identified the occupational therapy profession as being ideally positioned to contribute to public health agendas relating to tackling physical inactivity amongst marginalised populations, such as disabled people and people with experience of mental distress. However, a robust understanding of the enablers, restrictions, and exclusions such groups encounter when seeking to participate in sport and physical activity is currently lacking.

**Methods:**

This study aimed to gain an in‐depth understanding of the different ways people with experience of mental distress talked about their participation in a community‐based football league in England, in the UK. Nine people took part in this strand of a larger participatory action research (PAR) study, which used go‐along interviews as the method of data collection. In alignment with PAR seeking to address power imbalances, the data from the go‐along interviews were analysed through a Foucauldian lens using a collaboratively produced analytic framework.

**Findings:**

Participants constructed the community‐based football league as fostering feelings of purpose and belonging, against a backdrop of them describing experiencing stigma and exclusion when seeking to be active in their wider communities. They used the concept of occupational marginalisation to further interpret their situation.

**Conclusion:**

Understanding why and how people participate in football extends beyond seeing it as an individual exercise to shared social lives and occupations. With this perspective, occupational therapists could address occupational marginalisation in partnership with community sports organisations, collaborating for wider social change beyond specialist services.

**Key Points for Occupational Therapy**

- Participating in mainstream sport can be associated with discrimination, exclusion, and abuse for people with experience of mental distress, who perceive benefits from their involvement in community football projects.
- Occupational therapists can address experiences of occupational marginalisation in partnerships with players and coaches in community sports projects.
- These partnerships give scope for occupational therapy to engage with groups of marginalised people in community settings, working for broader social change in attitudes to mental distress.

## 1 INTRODUCTION

The global public health priority of physical inactivity (World Health Organisation, 2018) has close relevance to occupational therapy due to being concerned with the reciprocal relationship between what people do and their health and well‐being. Occupational therapy has been referenced in the Sport England workforce strategy as a profession that is uniquely positioned to contribute to sport and public health sector agendas to tackle physical inactivity (Sport England, 2018). This strategy emphasised inequality as a priority concern in addressing physical inactivity. Inequality has been exacerbated by the social and occupational restrictions imposed during the coronavirus pandemic. Marginalised populations such as disabled people and people with long‐term health conditions were unable to participate in community projects for prolonged periods (Sport England, 2021).

This paper reports part of a larger participatory action research (PAR) study which took place before the pandemic. It was conducted in collaboration with the Positive Mental Attitude (PMA) Sports Academy, a community‐based football league for people with experience of mental distress in England, in the UK. The football in this paper refers to the round ball form, also known as soccer. The project predominantly involved men identified as at risk, or with experience, of mental distress playing football together. The project was founded and run by an occupational therapist.

The definition of physical activity used within this paper and adopted by the World Health Organisation (2018) is “any bodily movement produced by skeletal muscles that results in energy expenditure” (Caspersen et al., 1985, p. 126). The term *physical activity* is preferred to *exercise* or *sport*, as it is more inclusive, encompassing occupations that are embedded in people's everyday lives, such as hanging up the washing and using a bicycle to commute, as well as defined sport and leisure occupations.

This paper seeks to position people with experience of mental distress as equal citizens. Therefore, the terms used to describe and state their experiences were discussed and agreed with the Research Steering Group that shaped this research study. The term *people with experience of mental distress* was chosen rather than descriptive biomedical terms such as psychiatric disorder, mental illness, or mental health problem. They chose this term because they intended it to be inclusive of all experiences, whether formally diagnosed or not, whether in receipt of secondary mental health services or not, and whether perceived as a current episode of mental distress or not. They also chose the term because they wanted to reflect that for some mental distress is a transitory experience, whereas a diagnosis or assignment of a mental health problem often feels like a permanent label. Furthermore, the paper uses the term *disabled people* to signal adherence with the social model of disability (Oliver, 2013).

People with experience of mental distress face inequalities and barriers to participation in physical activity, despite many wishing to be more active (Activity Alliance, 2021; Department for Culture Media and Sport, 2015; Sport England, 2021). These inequalities can be considered risk factors for occupational injustice such as occupational marginalisation, which arises when discrimination restricts choices and control in occupation (Hocking, 2017). This discrimination can be social, cultural, and political, where some perspectives are privileged over others. For example, sports opportunities associated with specialist mental health projects can be based on narrow, individualised, and biomedical understandings, with participation conceptualised as an adjunct to conventional mental health treatment and care (Friedrich & Mason, 2017b). By giving a central focus to treatment and individual symptoms, other aspects such as poverty, unemployment, and limited access to transport are marginalised. This narrow focus impacts on participation, arguably compounding and perpetuating occupational marginalisation in everyday life (Magee et al., 2015). Addressing inequality and marginalisation therefore requires broader social change (Morrow & Hardie, 2014).

Football (or soccer) can be an inclusive sport, with very little skew towards different ethnic groups, income groups, or educational status. It can therefore be viewed as having a potentially strong role in promoting occupational and social justice (Friedrich & Mason, 2017b; Magee & Jeanes, 2013). Football is described in the UK as the national game, engaging one in five adults (Friedrich & Mason, 2017a). However, despite a rapid growth in women's participation in recent years, in most countries, football is a mass participation sport played in the majority by men. To date, there has been a lack of occupation‐focused research that explores men's experiences of occupations, such as football (Hocking, 2012). This research study addresses that gap, with a particular focus on men with experience of mental distress.

The transactional relationship between playing football and the health and well‐being of people with experience of mental distress has been investigated (Dyer & Mills, 2011; Friedrich & Mason, 2017a, 2017b, 2018; Lamont et al., 2017; Mason & Holt, 2012; Mynard et al., 2009). These studies have demonstrated that playing football can have many benefits, including providing structure and purpose and improving physical health and mental well‐being (Carless & Douglas, 2008; Henderson et al., 2014; Hodgson et al., 2011; Moloney & Rohde, 2017; Such et al., 2019). Further benefits include distraction from experiences of mental distress, positive experiences of accomplishment, and the opportunity for a role shift from a mental health service user to a player (Dyer & Mills, 2011; Hodgson, 2012; Moloney & Rohde, 2017). However, the tendency for studies to be focused on the benefits and outcomes of participation obscures experiential knowledge of enablers, restrictions, barriers, and exclusions. Indeed, Spotswood et al. (2019) have suggested that physical activity needs to be reimagined as emerging from the way everyday social life is organised, rather than it being reduced to an outcome that arises from the dose or prescription of a specific intervention. For example, that difficulties getting out of the house and travelling on public transport will disrupt a disabled person from participating in activities at their local leisure centre. The need for such theorization and novel intervention approaches has recently been articulated in the occupational science literature (Bukhave & Creek, 2021). However, dominant within policy and literature is a representation of sport and physical activity that is framed by the medical model, exercise as medicine (Williams et al., 2018). This tendency fails to account for the complex social context, demands, and risks that participation entails (Pullen & Malcolm, 2018). Furthermore, it conceptualises physical activity as a single behaviour, rather than a set of diverse occupations that are embedded into people's everyday lives. Consequently, interventions have tended to be advanced on the basis that responsibility for change is ontologically situated with individuals through logics such as self‐care and choice (Pullen & Malcolm, 2018; Spotswood et al., 2019). Comparatively little attention has been paid to the social processes and structural factors that inevitably shape, limit, and restrict participation in various ways and perpetuate and compound existing inequalities (Williams & Gibson, 2018).

Investigating the impact of inequalities and experiences of marginalisation can be undertaken in different ways. Participatory research approaches can advance knowledge and understanding while simultaneously addressing inequality in the processes of knowledge production (Bryant et al., 2017). As co‐researchers, people with experience of mental distress are enabled to construct and make sense of their participation against dominant representation and ways of knowing. The outcomes of collaborative investigations can be useful for informing both policy and practice guidelines which shape therapeutic and public health interventions that seek to enable people with mental distress to participate in physical activity. For this reason, this strand of the study draws on an analytical framework that understands participation in the wider space of power relationships and intersecting discourses (Foucault, 1995).

The aim of the study was to explore the nature and value of participation in the PMA from the perspectives of those who took part. Other strands of the study have been reported elsewhere (Pettican et al., 2021).

## 2 METHODS

Those involved in the study were brought together as co‐researchers through a participatory action research (PAR) methodology. The impetus for the research study and its focus originated from the PMA, rather than from the researcher, and this set the scene for the research using a participatory approach. PAR can be defined as: “… a process in which ‘we’, researchers and participants, systematically work together in cycles to explore concerns, claims or issues that impact upon or disrupt people's lives” (Koch & Kralik, 2006, p. 27).

In enactment of the study's PAR methodology, a Research Steering Group was convened shortly after the study commenced. This was composed of both players and staff employed by the PMA (see Table 1 for details of Research Steering Group members) and the first author. The group agreed that collective discussion and decision‐making could occur in every stage of the research process.

**TABLE 1:** Research Steering Group members

Pseudonym (male/female)	Age	PMA role	Mental health care	Living arrangements
Sid (M)	Mid 50s	Player/coach	Secondary	Supported housing
Keith (M)^a^	Mid 30s	Player	Primary	Supported housing
Jalpesh (M)	Early 50s	Player	Secondary	With family
Tim (M) Joined Feb 2014	Mid 40s	Player	Primary	With family
Jake (M)^b^	Early 30s	Player	Secondary	With family
Donell (M)	Mid 30s	Player	Primary	Supported housing
Aaron (M)	Mid 30s	Player	Secondary	With family
Bret (M)	Mid 40s	Player	Secondary	Supported housing

^a^ Intermittent involvement due to fluctuations in health and circumstances.

^b^ Withdrew after meeting five due to family commitments.

### 2.1 Study design

The strand reported here developed the Research Steering Group's findings from earlier strands which revealed community and social aspects of participation in the PMA (Pettican et al., 2021). The group wanted to create opportunities for people to show as well as tell during the interview process. For this reason, go‐along interviews were agreed as the method of data collection (Castrodale, 2018; Garcia et al., 2012). Go‐along interviews involve the researcher meeting with the participant as an expert guide. As they travel together, the participant points out and explains significant details of the research setting. The researcher explores meanings and connections to the setting to develop understanding of the relationship between health and place. This collaborative process creates data which are contextual (Chang, 2016).

Go‐along interviews were felt to be particularly relevant given that the nature of participation in the PMA was synonymous with being outdoors and frequenting specific places and spaces (e.g. football training pitches, local community cafes, and the clubhouse). Importantly for this research study and its epistemology and methodology, go‐along interviews have also been identified as more democratic than conventional data collection methods. Knowledge can be co‐constructed, with participants shaping the direction (both literally and metaphorically) of the interview and therefore readdressing the power imbalance often inherent in research interviews.

#### 2.1.1 Participants

Data were collected by the first author, an occupational therapist, via 11 go‐along interviews with nine individuals (one of the interviews was conducted with a pair, and three people were interviewed twice). Those who took part were involved in the PMA in some way (e.g. as a player or coach). Therefore, the study used purposive sampling. The opportunity to participate in the go‐along interviews was communicated via a flyer, which was posted to the PMA website and read out at preceding training sessions and match days. Although participants were required to be able and willing to give informed consent, their engagement in the go‐along interview was flexible, and therefore, one interview took place in a local community café. Details of the participants are provided in Table 2. Five of the Research Steering Group members were participants in the go‐along interviews. The sample included six PMA players and three past and present members of staff. Seven men and two women, with an age range of 34–55. There were no refusals or dropouts.

**TABLE 2:** Go‐along interviewees

Name (male/female)	Age	PMA role	Mental health care	Living arrangements
Sid (M)^a^	Mid 50s	Player/coach	Secondary	Supported housing
Bret (M)^a^	Mid 40s	Player	Secondary	Supported housing
Jalpesh (M)^a^	Early 50s	Player	Secondary	With family
Donell (M)^b^	Mid 30s	Player	Primary	Supported housing
Aaron (M)^b^	Mid 30s	Player	Secondary	With family
Kate (F)	Mid 40s	Staff	N/A	N/A
Jamie (F)	Early 40s	Staff	N/A	N/A
Dwayne (M)	Late 30s	Player/staff	GP	Independent
Keith (M)	Mid 30s	Player	GP	Supported housing

^a^ Participants who returned to participate in repeat interview.

^b^ Participants who chose to be interviewed together.

#### 2.1.2 Ethical considerations

The Research Steering Group ensured that the research was planned and carried out in an ethical manner. By negotiating, agreeing, and planning each stage together, emerging understandings of the study, research process, and topic remained relevant to their lives and values.

Research ethics approval was obtained from the School of Health and Social Care at the University of Essex (Ref 13010 and 13010a). The Research Steering Group worked together to develop the documentation relating to recruitment and consent. Involving people in designing a study's consent process can enhance its relevance and accessibility (Involve, 2012).

#### 2.1.3 Data collection and analysis

Participants kept within a geographical area associated with their participation in the PMA, but ultimately each participant decided the pace and route of their interview. The interviews were recorded on small recorders that could be put into a pocket or discreetly clipped on to clothing. The first interview acted as a pilot but went well with little adjustment to the interview schedule, so was included in the analysis. Field notes were made after each interview. All interviews were recorded and fully transcribed. MAXQDA was used as a tool to organise and code data.

#### 2.1.4 Creating the analytic framework and data analysis

The Research Steering Group maintained a clear role in directing and informing the data analysis stage of the study by creating an analytic framework together. This approach to data analysis was informed by the work of Foucault, who discouraged formalisation of the analytical concepts and tools associated with his work around discourse analysis. Instead, he advocated that the needs of the study would best guide use of these concepts and tools. Discourse analysis can be understood as having a primary focus on *how* things are said, rather than *what* is said, as is conventionally the case in thematic analysis. It seeks to reveal power relations in society and how certain practices and positions are rendered desirable and possible (Springer & Clinton, 2015). There is a tendency for discourse analysis studies to not involve participant validation, let alone other forms of involvement (Harper, 2003, 2008). Such discussions highlight some of the practical and ethical complexities of asking participants to validate their position within discourse(s) when this may not be something they are consciously aware of. However, discourse analysis can be used as a tool for social action (Willig, 1999), which would move it beyond discussions about the potential of participant validation, to more collaborative forms of research, as in the case of this study.

The Research Steering Group constructed the analytic framework by combining the headings from a literature review the first author had undertaken, the themes from the first strand (Pettican et al., 2021), and records of their discussions. They started at a micro level of statements and discourses concerned with, for example, limited organisational resources. Then they gradually worked upwards (or synthesised the material into) and agreed three distinct discourses. Developing the analytic framework was not a neat linear process, but rather it was an iterative one involving many discussions, revisions, and refinements. The three distinct discourses, identified because they had greatest relevance to the agreed research question and focus for this research study, were:

1. Participation as *healthy*
2. Participation as *social*
3. Participation as *occupational*

The transcripts and discourses of the research participants were then mapped against the analytic framework to identify ways in which they drew from, or resisted, the three identified discourses.

Although there is necessarily some linearity in its presentation here, the analysis process was messy and iterative, as the Research Steering Group worked together in cycles to explore the transcripts against the analytic framework. This process enabled each discourse to be better understood and for illustrative quotes to be identified and agreed. Although the three discourses will be outlined and developed separately, it should be noted that the discourses are interdependent, existing of and through each other. One discourse can to a degree be seen to be defined by or through another. All transcripts were analysed and included, and data saturation was achieved, as no new insights emerged from the last interviews.

## 3 FINDINGS

The findings relating to each discourse are detailed in this section. The findings make use of Foucault's concept of disciplinary power, which asserts that power is dispersed and pervasive (Foucault, 1995), in terms of how it might regulate and limit participation, marginalising people in sustained and various ways.

### 3.1 Participation as healthy

The first discourse is *participation as healthy*, which has its roots in the medical model and constructs participation in sport and physical activity as health enhancing. The fit and active body is healthy, and the unfit and inactive body is unhealthy. This discourse dominates public health, mental health, and sport policy, positioning people and directing how and where they participate. Several of the participants connected their participation in the PMA to weight loss and constructed themselves as needing to lose weight, highlighting a potential motivator for participation. Jalpesh is a long‐term user of mental health services and PMA player:

Extract 1: Jalpesh (second strand go‐along interview)

273
: *“Well my health like I said before have improved a lot from day*
274
: *one about 100% I lost 2.5 stone so was 12 stone now I'm 10.5*
275
: *um have become more like I said um more active my mind my*
276
: *body my soul and spirit I become more more mobile …”*

However, there were also counter illustrations of how the side effects of psychiatric treatment might limit and restrict people with mental distress when trying to participate in a sport such as football. Dwayne's comparison with someone who is not taking such medication might also suggest why participation in mainstream sports might be difficult. Dwayne was originally a player within the PMA but later became a staff member, as well as being a long‐term user of inpatient and community mental health services:

Extract 50: Dwayne (second strand go‐along interview)

638
: *“when you are*
639
: *taking heavy um psychiatric medication it can it can the effects*
640
: *can slow you down you are you are your abilities is not the same*
641
: *as someone who's not taking it you know not for everyone but*
642
: *you know for the most people so you are at a disadvantage*
643
: *already so to be able to get fit in somewhere where the*
644
: *individuals they are not taking it and you are you are facing*
645
: *another challenge”*

The complexity of participation, and the limited choice and control people with experience of mental distress encounter in relation to their everyday occupations, is also outlined by Sid. He talks about how the requirement to have a regular depot injection (a slow‐release form of psychiatric medication) sometimes limited his participation in the PMA as it requires him to leave at a certain time:

Extract 9 Sid (third strand go‐along interview)

627
: *“… sometimes on a*
628
: *Thursday like when the game is going on for a little longer I*
629
: *have to leave early because every four weeks I get my depot*
630
: *and everyone is used to that now I try not to go …*

Thus, there are tensions within this discourse that illuminate that while being active and playing sport might be encouraged by the psychiatric system, the side effects of psychiatric treatment and requirements to attend regular medication appointments can also limit participation to specialist projects, where they will be an understanding and acceptance of such effects and requirements.

### 3.2 Participation as social

The second discourse is *participation as social*, which has its roots in the social model of disability, as a response to the limitations associated with the medical model. This views disability as arising from the way society is organised and structured to marginalise some people and privilege others, rather than from a person's condition or difference. Bret talks about the importance he attaches to everyone who participates in the PMA having a shared experience of mental distress, as it fosters belonging against a backdrop of the discrimination and exclusion he encounters in wider society. Bret is a PMA player with a long history of involvement in mental health services:

Extract 13: Bret (third strand go‐along interview)

319
: *Anna: “Was it important for you though that the other*
320
: *players had mental health … was that helpful in some way?”*
321
: *Bret: “No that was very important very important”*
322
: *Anna: “Can you say why was that important for you?”*
323
: *Bret: “Well because um very important because first of all you are*
324
: *relating to other people like it's part of belonging there's a*
325
: *sense of belonging because you have all been through similar*
326
: *problems you know but like before PMA or before Arsenal in*
327
: *the Community project um I could not play football because if*
328
: *you just play football you know just sort of so‐called normal*
329
: *members of society basically strangers first of all I do not know*
330
: *them and secondly because of my condition that it is very*
331
: *rarely that people are sympathetic to your condition because*
332
: *they are just probably be afraid or they think you are weird or*
333
: *something like that and it causes problems you know and I*
334
: *could easily get into fights so I just avoided going to the park I*
335
: *would not play football in fact I could not play any kind of sports*

Here, Bret problematises his experience of mental distress when seeking to play sport in mainstream settings, with the PMA positioned in contrast as fostering feelings of belonging due to players' shared experience of mental distress. A sense of the PMA providing a space of refuge is mirrored in Keith's extract:

Extract 17: Keith (third strand go‐along interview)

255
: *“I like football I like the buzz of it I like the guys I play with I*
256
: *like the guys we play against I like there's there's there's no*
257
: *judgement I like to be in an environment where there is*
258
: *minimal judgement like I cannot work through a market or even a*
259
: *high road from my house without someone attacking me*
260
: *somewhat because I'm that little bit more eccentric than the*
261
: *society around do you know what I mean so like here I fit in I*
262
: *slot in …”*

This discourse therefore provides a sense of the discrimination that people with experience of mental distress encounter when seeking to play sport in mainstream settings, due to potential abuse. It also highlights belonging as an enabler.

### 3.3 Participation as occupational

The third discourse is *participation as occupational*. A pattern that emerged consistently over the analysis of all the interviews was that it was helpful to see football as something to do in its broadest sense. Keith talks about his participation in the PMA and it being transformational in terms of keeping him out of hospital. Interestingly, in contrast to the participation as healthy discourse, he does not make a connection here with the PMA being an organisation that is concerned with working therapeutically with people with experience of mental distress. Rather, he positions it as a separate experience that positively dilutes the general focus in his life on his mental health:

Extract 40: Keith (third strand go‐along interview)

53
: *“I've never dropped off unless I've been unless I had a injury to*
54
: *deal with or my mental health deteriorated where I had to go*
55
: *into hospital and stuff I never really had an admission since the*
56
: *football the football is probably one of the things that has*
57
: *helped me keep out of hospital because it's given me*
58
: *something positive to focus on rather than my mental health*
59
: *dominating my life and stuff …”*

Participation in football acting as an important leveller for people with experience of mental distress is also illustrated in the quote from Jamie. Jamie is the founder of the PMA, and she describes her impression of how it can enable an alternative sense of self and foster connections and commonality with wider society:

Extract 48: Jamie (third strand go‐along interview)

523
: *“Yeah exactly and then they can watch it on the telly and be*
524
: *part of it because everyone watches football on the telly and*
525
: *[…] everyone puts the top on and*
526
: *goes to football and now they feel as though they are part of*
527
: *the community again … I've got my Arsenal top on and I'm*
528
: *playing football like he does over the road and he is in a club*
529
: *and you know he is not mental health and I am but now I am*
530
: *doing what he is doing and then it is like accepting them in the*
531
: *community they feel as though they are part of it again*

## 4 DISCUSSION

The three discourses demonstrate the different ways in which people with experience of mental distress might construct their participation in sport and physical activity, with explanation and illustrative quotes derived from the collective analysis process within the Research Steering Group.

The findings support previous research that has highlighted the various benefits and outcomes that can be derived from people with experience of mental distress playing football (Carless & Douglas, 2008; Henderson et al., 2014; Hodgson et al., 2011; Moloney & Rohde, 2017; Such et al., 2019). However, the study provides new knowledge about factors that might enable participation and mediate the benefits that are derived. For example, that specialist sports projects, such as the PMA, provide a safe place around the shared experience of mental distress, which fosters feelings of acceptance and belonging. These findings support calls for research that develops understanding about how participation in sport and physical activity is best organised for marginalised populations, such as people with mental distress, to promote participation and maximise the benefits that are derived (Department for Culture Media and Sport, 2015; Sport England, 2021).

The findings also detail several factors that might restrict people with experience of mental distress from playing football in mainstream settings, such as the threat of discrimination and abuse. Findings relating to the further limits that can arise from the side effects of medication align with previous research, which found that lethargy and weight gain were the main challenges for people with mental distress who wanted to play sport as part of an organised physical activity programme (Hodgson et al., 2011). Occupational marginalisation, as a risk factor for injustice, was discussed by the Research Steering Group during the analysis process and agreed as a useful way of interpreting these findings, in terms of participants describing the stigma and discrimination they encounter in their everyday lives (Hocking, 2017). As a conceptual lens, occupational marginalisation aligns with limits to choices and control over physical activities in everyday life, rather than reducing participation to a single behaviour (Spotswood et al., 2019).

This new knowledge could enhance occupational therapists' specialist skills in identifying access and performance problems and enabling participation. Therefore, there is much the profession could contribute to addressing physical inactivity among marginalised populations (Sport England, 2018, 2021). However, these findings also suggest the need to promote occupational justice by working towards wider social change and working with sports coaches and other professionals to make use of the UK's Mental Health Charter for Sport and Recreation (Sport and Recreation Alliance, 2015). This charter supports sport and recreation organisations to adopt good mental health practices to make activities inclusive, positive, and open to everyone. Occupational therapists should consider working pro‐actively with national governing bodies of sport, as social institutions who influence who gets to play sport and on what terms. Rather than being solely focused on therapeutic work with individuals, occupational marginalisation could then be effectively addressed by changing public attitudes and behaviour towards people with experience of mental distress in mainstream settings.

This study extends previous occupational therapy research that has explored participation in round ball football from the perspective of it being an adjunct to statutory mental health treatment focused on people with experience of mental distress (Moloney & Rohde, 2017). Indeed, an occupational justice perspective acts a valuable counter to health and sport policies, which are often overly reliant on individual‐level behaviour change (Pullen & Malcolm, 2018; Williams & Gibson, 2018). The use of a PAR approach ensures that knowledge is advanced in directions that are relevant and meaningful to people with experience of mental distress. However, there is still a lack of research that explores in depth the factors that influence people to give up participating in sport and other physical occupations and addressing this knowledge gap should be a priority for the future. Such research would enable more precise support to be offered where it is needed, which previous research has identified as critical in enabling and sustaining participation (Hodgson et al., 2011).

### 4.1 Limitations

Several limitations are acknowledged in relation to this research study. Similarly, to previous research involving football projects for people with experience of mental distress, this study involved only one female participant who was a player within the PMA. Further research in football or other sports that might be more appealing to women is necessary to investigate their participation, nature, and value more fully.

It is a limitation that the nature of go‐along interviews necessitates that people feel comfortable in public spaces, when having difficulty with this might be part of people's experience of mental distress. However, the option of conducting the interview elsewhere was made, and in one instance taken up (the interview was conducted in a community café). Furthermore, there are risks to spatial confidentiality if other people are encountered during the interview. This was managed by the first author and participants agreeing what would be said prior to starting the interview if they encountered someone they knew.

## CONFLICT OF INTEREST

The authors have no conflict of interest to declare.

## AUTHOR CONTRIBUTIONS

All five authors made substantial contributions to the conception and design of the study. AP gathered the data. AP facilitated data analysis with members of the Research Steering Group and input from all the other authors. AP wrote the first draft of the paper with revision and input from all the other authors.

## Data Availability

The data that support the findings of this study are openly available in the University of Essex Research Repository at http://repository.essex.ac.uk/24364/.
